# Simultaneous Systemic Lupus Erythematosus Flare and Disseminated Tuberculosis: Balancing Anti-Mycobacterial and Autoimmune Treatments

**DOI:** 10.7759/cureus.18944

**Published:** 2021-10-21

**Authors:** João E Silva, Clara Silva, Mariana Pacheco, Edite Pereira, Jorge S Almeida

**Affiliations:** 1 Internal Medicine, Centro Hospitalar Universitário de São João, Porto, PRT; 2 Medicine, Faculdade de Medicina da Universidade do Porto, Porto, PRT

**Keywords:** immunosuppression, systemic lupus erythromatosus, disseminated tuberculosis, anti-tuberculosis treatment, lupus flare

## Abstract

Here, we report the case of a 53-year-old man with suspected autoimmune arthritis on low-dose corticosteroid therapy. He was recently hospitalized due to presumed bacterial pneumonia and a seizure episode attributed to high fever. His condition deteriorated after discharge, and he presented to our institution with a persistent cough, weight loss, skin rash, arthralgias, fever, and altered mental status. The investigation led to the simultaneous diagnosis of a systemic lupus erythematosus (SLE) flare and disseminated tuberculosis (TB), both pulmonary and intracranial. Proteinuria and peripheral edema were identified, suggesting renal involvement of SLE. Anti-mycobacterial drugs and high-dose corticosteroid therapy were initiated. Given the risk of starting other immunosuppressive drugs in the presence of intracranial TB, in a patient with stable renal function and a significant decrease in proteinuria with corticosteroids and supportive therapy alone, renal biopsy was postponed. Prednisolone was progressively tapered down during the next six months, always maintaining anti-mycobacterial therapy, which resulted in a second SLE flare and the need to increase corticosteroids again. At this time, a renal biopsy was performed, showing class II lupus nephritis and confirming the diagnosis of SLE. After one year of anti-mycobacterial therapy with complete resolution of cerebral and pulmonary TB lesions, we chose to initiate mycophenolate mofetil as an immunosuppressive steroid-sparing agent with increased SLE control, allowing for corticosteroid reduction.

## Introduction

Systemic lupus erythematosus (SLE) is a chronic autoimmune disease characterized by its multisystemic manifestations. Infections contribute to the burden of morbidity and mortality in SLE and represent the main cause of early death in patients [1,2]. The higher incidence of infections in patients with SLE results from the immunosuppressive agents used in the treatment and the impaired immune response associated with SLE [3,4]. The use of immunosuppressive agents, flares, high disease activity, and the presence of lupus nephritis are among the most important risk factors for the development of infections in SLE patients [5-8].

Interaction between SLE flares and infection is complex. They may occur simultaneously because infections may trigger disease flares and disease flares predispose patients to infections [3,4]. Respiratory, urinary, and soft tissue bacterial infections are the most common infectious complications in SLE [6,7]. However, in countries with a higher prevalence, the risk of opportunistic infections such as tuberculosis (TB) must be considered, with several studies showing an increased predisposition to TB infection among SLE patients [9,10]. Given their impaired immunity, presentations with severe pulmonary and extrapulmonary TB are more common in SLE patients than in the general population [10].

When both an SLE flare and an infection occur, physicians face the need to balance the use of immunosuppressive agents to control the flare with the risk of those agents impairing the immune system response to an active infection. In infections such as TB, when prolonged antibiotic treatment is needed, caution must be practiced regarding the risk for new SLE flares while immunosuppressive therapies are kept to a minimum.

## Case presentation

Here, we report the case of a 53-year-old caucasian man who presented to our institution with a persistent cough, weight loss, skin rash, arthralgia, fever, and altered mental status.

The patient had visited another hospital due to polyarthralgia one year before. Autoimmune disease was suspected and he was started on prednisolone, progressively tapered down. However, because he was lost to follow-up, no definite diagnosis was made. The patient was not vaccinated with Bacille Calmette-Guérin as a child. Furthermore, a purified protein derivative skin test or gamma-interferon assay was not performed prior to initiating corticosteroid therapy. Other recent medical history involved two hospital admissions. The first, eight months before, was due to right inferior lobar pneumonia, which was treated with amoxicillin plus clavulanic acid. In the second admission, one month before presenting to our institution, he was again admitted with respiratory symptoms and conventional radiography findings compatible with right inferior lobar pneumonia. The in-hospital stay was complicated with a seizure episode attributed to high fever. A brain computerized tomography (CT) scan performed at the time showed no abnormalities. He was treated with levofloxacin, and his symptoms improved temporarily. However, after two weeks, his condition deteriorated once again, which prompted the visit to our hospital. Other medical history was irrelevant. In addition to prednisolone 10 mg daily, the patient was medicated with omeprazole OD and levetiracetam BID.

On admission, the patient was hemodynamically stable but febrile. He showed signs of confusion, with temporal and space disorientation, and was unable to provide accurate anamnesis by himself. A full neurological examination revealed left-sided hemiparesis and ataxia. Skin inspection revealed cutaneous rash and peripheral edema. Although he complained of arthralgia, there were no signs of arthritis. Pulmonary auscultation showed bilateral cutaneous crackles and rhonchi, without hypoxia. The remaining anamnesis and physical examination did not reveal any additional findings. The initial laboratory workup showed elevated C-reactive protein of 66.7 mg/dL (normal values <5 mg/dL); elevated sedimentation rate of 65 mm/hour (normal 0-15 mm/hour) without leucocytosis; normocytic, normochromic anemia (hemoglobin 11.7 g/dL); and elevated lactate dehydrogenase of 440 U/L (normal range 140-280 U/L). There was no evidence of cholestasis, hyperbilirubinemia, or hepatic cytolysis. Creatinine and urea were within the normal range, but hypoalbuminemia of 29.3 g/L (normal range 38-51 g/L) and proteinuria of 2.5 g/L (normal values <0.15 g/L) were detected. His human immunodeficiency virus test was negative. A summary of laboratory findings is shown in Table 1.

**Table 1:** Summary of laboratory findings. ADA: adenosine deaminase; ALP: alkaline phosphatase; ALT: alanine aminotransferase; ANAs: anti-nuclear antibodies; ANCA: anti-neutrophil cytoplasmic antibodies; anti-CCP: anti-cyclic citrullinated peptides antibodies; anti-dsDNA: anti-double-stranded DNA antibodies; AST: aspartate aminotransferase; C3: conjugated complement component 3; C4: complement component 4; CRP: C-reactive protein; CSF: cerebrospinal fluid; ENA: extractable nuclear antigens; ESR: erythrocyte sedimentation rate; FT4: free thyroxine; GGT: gamma-glutamyl transferase; PCR: polymerase chain reaction; RF: rheumatoid factor; TSH: thyroid-stimulating hormone

Parameter	Value	Ref. value	Parameter	Value	Ref. value
Hemoglobin	11.7 g/dL	12.0–16.0	Total bilirubin	0.78 mg/dL	<1.2
Leucocytes	5.93 × 10^9^/L	4.0–11.0	Direct bilirubin	0.32 mg/dL	<0.4
Platelets	252 × 10^9^/L	150–400	Glucose	106 mg/dL	75–110
CRP	13.9 mg/L	<5.0	Total cells (CSF)	96/μL	
ESR	65 mm/1^st^ hour	0–20	Leukocytes (CSF)	55.2 /μL	<4
Creatinine	0.71 mg/dL	0.51–0.90	Total proteins (CSF)	1.29 g/L	0.15–0.45
Urea	50 mg/dL	10–50	ADA (CSF)	13 U/L	
Sodium	136 mEq/L	135–147	ANAs	>1/1,000	<1/100
Potassium	3.8 mEq/L	101–109	Anti-dsDNA	384.1	<100
Chloride	105 mEq/L	64–83.0	ANCA panel	Negative	
FT4	1.17 ng/dL	0.7–1.48	ENA antibodies panel	Negative	
TSH	1.58 μgUI/mL	0.35–4.94	RF	<8.9 UI/mL	<30
ALT	9 U/L	10–31	Anti-CCP	1.7 U/L	<7
AST	18 U/L	10–31	C4	4 mg/dL	16–48
GGT	26 U/L	7–32	C3	32 mg/dL	80–160
ALP	38 U/L	30–120	*Mycobacterium tuberculosis* (PCR, sputum)	Positive	
Albumin	29.3 g/L	38-51			
Total proteins (urine)	2.5 g/L	<0.15			

**Figure 1:**
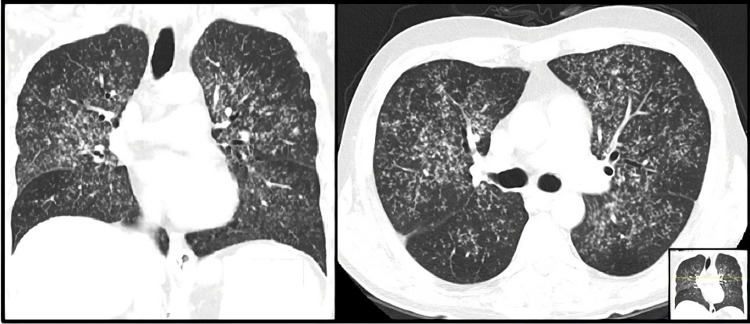
Coronal and axial images of the first thoracic CT scan showing innumerable, small 1-4 mm pulmonary nodules scattered throughout the lungs, a pattern suggestive of miliary TB. CT: computed tomography; TB: tuberculosis

**Figure 2:**
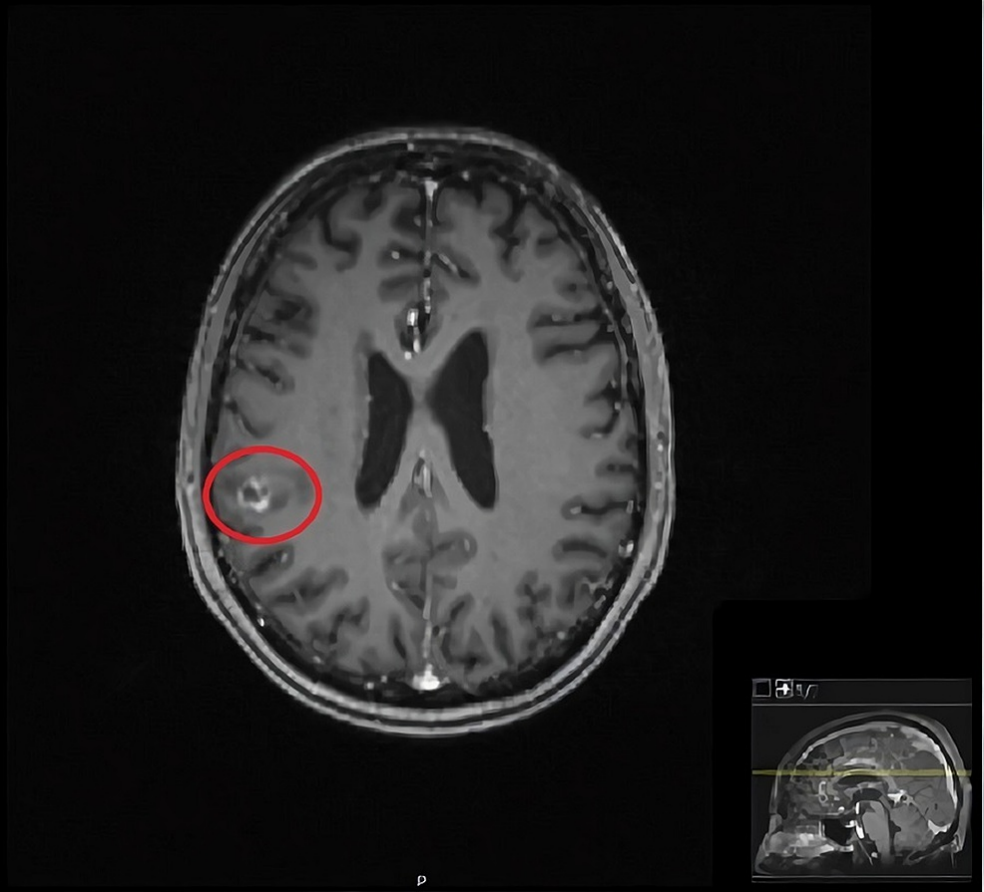
Cerebral MRI showing an intracranial tuberculous granuloma (red circle), with a ring of peripheral enhancement on T1-weighted contrast-enhanced image. MRI: magnetic resonance imaging

To clarify the recent pulmonary infections and altered mental status, further investigation was performed. Thoracic CT scan revealed a pattern suggestive of pulmonary miliary tuberculosis (Figure 1), and *Mycobacterium tuberculosis* was readily detected in sputum samples by a DNA polymerase chain reaction assay. This result was confirmed by culture growth after 12 days. A lumbar puncture test showed cerebrospinal fluid pleocytosis, elevated proteins, elevated glucose, and elevated adenosine deaminase (13 U/L). Brain magnetic resonance imaging (MRI) showed lesions compatible with intracranial TB (Figure 2). The presence of rash, arthralgias, anemia, and proteinuria could not be explained by TB. Given the history of suspected autoimmune disease, complement and autoimmune tests were performed, which showed hypocomplementemia and elevated anti-nuclear antibodies and anti-double-stranded DNA antibodies (anti-dsDNA), raising the suspicion that these symptoms were secondary to a concomitant SLE flare. Antiphospholipid antibodies, including lupus anticoagulant, anti-cardiolipin antibodies, and anti-β2-glycoprotein I antibodies, were negative, ruling out the presence of secondary anti-phospholipid syndrome.

The patient was started on quadruple anti-mycobacterial therapy and prednisolone tapered up to 1 mg/kg daily. After three weeks of hospital stay, the patient showed almost full neurological recovery and complete resolution of anemia, arthralgia, and skin rash. Proteinuria decreased significantly, and hypoalbuminemia and edema also improved. The patient was discharged on prednisolone 1 mg/kg daily and anti-mycobacterial therapy. Renal biopsy was considered to fully confirm the SLE diagnosis and stage kidney disease but was postponed because the flare was controlled and proteinuria subsided on corticosteroids without the need for further immunosuppression.

**Figure 3:**
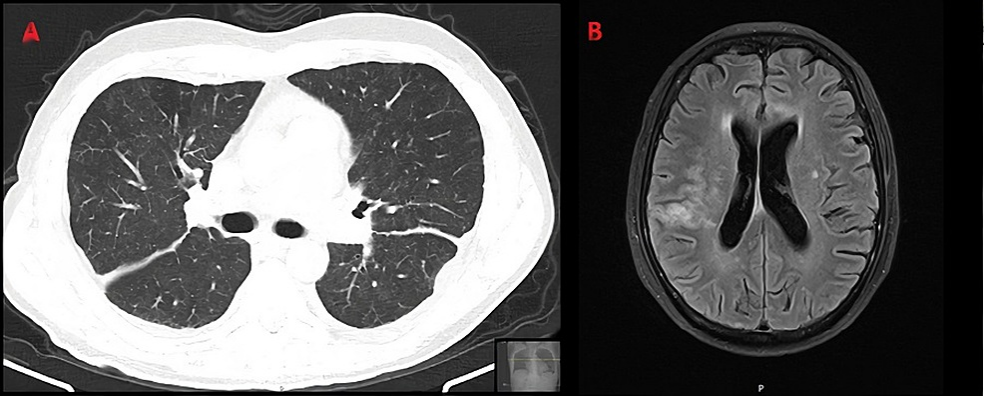
Repeat thoracic CT scan (A) and cerebral MRI (B) showing favorable evolution of the TB lesions. CT: computed tomography; MRI: magnetic resonance imaging; TB: tuberculosis

Prednisolone was tapered down over a six-month period, reaching a dose of 5 mg/day. At this time, the patient presented with a new SLE flare, characterized clinically by fatigue, malaise, peripheral edema, foamy urine, cutaneous rash, and analytically by hemolytic anemia (hemoglobin 9.7 g/dL, positive Coombs’ test), thrombocytopenia (36 × 10^9^/L), aggravated hypoalbuminemia and proteinuria, increase in anti-dsDNA titers, and complement consumption. A kidney biopsy was performed revealing class II lupus nephritis, with intermediate signs of chronicity and activity. Pulmonary CT scan and brain MRI were repeated, which showed complete resolution of the pulmonary TB lesions and only residual edema at the site of intracranial TB lesions seen previously (Figure 3).

The patient was restarted on prednisolone 1 mg/kg/day with rapid flare control. The patient was maintained on prednisolone tapered down to a minimum of 20 mg/day till he stopped anti-mycobacterial therapy after one year of treatment (three months of quadruple therapy, followed by nine months of isoniazid and rifampicin). Subsequently, he was started on mycophenolate mofetil (MMF) tapered up to 1,000 mg BID with improved SLE control, allowing for prednisolone reduction (<7.5 mg OD).

## Discussion

Patients with SLE are susceptible to infections due to immunosuppressive therapies and immune system abnormalities, including immunoglobulin and complement deficiencies, defects in chemotaxis, phagocytosis, delayed hypersensitivity, and abnormalities of cellular immunity [3,5,8]. Several studies show a greater incidence of TB in SLE patients compared to the general population, with more advanced disease forms leading to impaired immunity, including pulmonary miliary forms and extrapulmonary TB [9,10].

Balancing immunosuppressive therapy is vital during simultaneous SLE flare and active infection, especially in the presence of severe infections such as disseminated TB. Some studies have correlated the use of higher doses of corticosteroids with higher mortality when these conditions occur simultaneously [11]. Studies concerning other immunosuppressive agents are scarce, but retrospective studies with intravenous immunoglobulin have shown good efficacy and safety profiles during severe SLE flares and active infection [12,13]. Nevertheless, SLE patients with extrapulmonary TB have higher mortality, especially in central nervous system (CNS)/meningeal TB [1,2,14].

There are no specific guidelines for the treatment of TB in patients with SLE or vice versa. The optimal strategy is screening for latent TB and treating it when detected (preferably with a nine-month isoniazid-based regimen) in all patients with SLE, or other rheumatic diseases requiring immunosuppression, and waiting at least four to eight weeks before initiating immunosuppressive therapy [15,16]. In the presence of active TB in patients with SLE or other rheumatic diseases, treatment follows the same recommendations for combination therapy as in other patients, with a preference for initial intensive treatment with quadruple therapy for at least two months, followed by at least four months of double therapy with isoniazid plus rifampicin and/or ethambutol. This treatment phase can be extended for three to six months among patients with diabetes, those with radiographic cavities, extensive extrapulmonary diseases, or in those who remain smear- or culture-positive at the end of the intensive phase [17,18]. Biological treatments should be suspended for at least six months when possible [15]. Adjuvant corticosteroid therapy is not usually indicated in active TB except pericardial and intracranial TB, where corticosteroids show lower mortality [19].

There is no specific guidance on simultaneous SLE flare and active disseminated TB. The literature consists mostly of case reports. The most common approach is the single use of corticosteroids as an immunosuppressive agent in combination with anti-mycobacterial treatment. A retrospective study by Cheng et al. [11] correlated higher prednisolone doses during TB treatment and cyclophosphamide pulses before TB diagnosis with higher mortality in SLE patients. However, these findings were from a cohort with multiple forms of pulmonary and extrapulmonary TB, where pericardial TB was not studied separately. Furthermore, after multivariate analysis, corticosteroid usage showed a tendency to reduce mortality in patients with CNS involvement by TB (hazard ratio = 0.64, 95% confidence interval = 0.07-5.43) [11].

In our case, the decision was taken to start quadruple anti-mycobacterial therapy (rifampicin, isoniazid, pyrazinamide, and ethambutol) in combination with prednisolone 1 mg/kg/day, given its proven benefit in both intracranial TB and SLE flare control. Our prednisolone dosage was lower than the equivalent dexamethasone dosage used by Thwaites et al. for meningeal TB [20]. We chose this lower dosage to minimize interference with the efficacy of anti-mycobacterial drugs because our patient was not in a coma and did have any focal signs which could justify full corticosteroid dosage for intracranial TB; moreover, the SLE flare was not severe enough to prompt heavier immunosuppression. In more severe flares with simultaneous active infection, intravenous immunoglobin should be considered, with retrospective studies showing favorable outcomes [12,13].

After one year of anti-mycobacterial drugs, a corticosteroid-sparing agent (MMF) was chosen as maintenance therapy for lupus nephritis. By then, both cerebral MRI and chest CT scans showed complete resolution of the TB lesions. MMF was preferred due to its positive results on the initial and subsequent treatment of lupus nephritis and its low toxicity profile [21].

Unfortunately, after another nine months, the patient was readmitted with multifocal ischemic stroke, which was later assumed to be cerebral SLE vasculitic involvement (due to new increase in anti-dsDNA titers, complement consumption, CSF analysis not suggestive of infection, and cerebral MRI showing multiple sites of arterial vasculitic stenosis in different territories associated with focal ischemia). MMF was suspended and the patient was started on rituximab. Rituximab is considered safe in this setting, with several studies showing an almost negligible risk of TB reactivation in rheumatologic patients treated with rituximab [22]. The patient completed two doses of rituximab (1,000 mg) and remained free of SLE flares and without signs of TB reactivation after six months of follow-up. The patient maintains therapy with prednisolone 7.5 mg OD, hydroxychloroquine 400 mg OD, and proceeded with the third administration of 1,000 mg of rituximab.

## Conclusions

Infectious complications contribute to the burden of morbidity and mortality in SLE and represent the main cause of early death in these patients. Opportunistic infections such as TB must be considered, given the increased predisposition to TB infection among SLE patients.

This case illustrates the difficulty in managing concomitant active SLE and disseminated TB. An effort must be made to balance the use of immunosuppressive agents to control the flare with the risk of those agents impairing the immune system response to active TB infection and contribute to the emergence of mycobacterial antibiotic resistance. More studies are required to establish formal guidelines for the management of these patients.
